# Oscillatory behavior of ventricular action potential duration in heart failure patients at respiratory rate and low frequency

**DOI:** 10.3389/fphys.2014.00414

**Published:** 2014-10-28

**Authors:** Ben Hanson, Nick Child, Stefan Van Duijvenboden, Michele Orini, Zhong Chen, Ruben Coronel, Christopher A. Rinaldi, Jaspal S. Gill, Jaswinder S. Gill, Peter Taggart

**Affiliations:** ^1^Department of Mechanical Engineering, University College LondonLondon, UK; ^2^Cardiovascular (Imaging) Department, King's College LondonLondon, UK; ^3^Institute of Cardiovascular Science, University College LondonLondon, UK; ^4^Department of Experimental Cardiology, Academic Medical CenterAmsterdam, Netherlands; ^5^Division of Medicine, University College LondonLondon, UK

**Keywords:** action potential duration, Mayer wave, respiration, oscillation, ARI

## Abstract

Oscillations of arterial pressure occur spontaneously at a frequency of approximately 0.1 Hz coupled with synchronous oscillations of sympathetic nerve activity (“Mayer waves”). This study investigated the extent to which corresponding oscillations may occur in ventricular action potential duration (APD). Fourteen ambulatory (outpatient) heart failure patients with biventricular pacing devices were studied while seated upright watching movie clips to maintain arousal. Activation recovery intervals (ARI) as a measure of ventricular APD were obtained from unipolar electrograms recorded from the LV epicardial pacing lead during steady state RV pacing from the device. Arterial blood pressure was measured non-invasively (Finapress) and respiration monitored. Oscillations were quantified using time frequency and coherence analysis. Oscillatory behavior of ARI at the respiratory frequency was observed in all subjects. The magnitude of the ARI variation ranged from 2.2 to 6.9 ms (mean 5.0 ms). Coherence analysis showed a correlation with respiratory oscillation for an average of 43% of the recording time at a significance level of *p* < 0.05. Oscillations in systolic blood pressure in the Mayer wave frequency range were observed in all subjects for whom blood pressure was recorded (*n* = 13). ARI oscillation in the Mayer wave frequency range was observed in 6/13 subjects (46%) over a range of 2.9 to 9.2 ms. Coherence with Mayer waves at the *p* < 0.05 significance level was present for an average of 29% of the recording time. In ambulatory patients with heart failure during enhanced mental arousal, left ventricular epicardial APD (ARI) oscillated at the respiratory frequency (approximately 0.25 Hz). In 6 patients (46%) APD oscillated at the slower Mayer wave frequency (approximately 0.1 Hz). These findings may be important in understanding sympathetic activity-related arrhythmogenesis.

## Introduction

Oscillatory activity is a ubiquitous property of autonomic nerves innervating the heart, and is considered by some to facilitate synchronization of nerve traffic and hence potentiate the response. Oscillations at a low frequency of approximately 0.1 Hz, known as Mayer waves, occur in arterial pressure coupled with synchronous sympathetic efferent nerve activity and are exaggerated during enhanced sympathetic activity (Julien, 2006; Malpas, 2010). The mechanism of these systolic blood pressure oscillations is thought to involve sympathetic modulation of peripheral vascular resistance and the baroreflex response, although their exact relationship remains to be determined (Julien, 2006; Malpas, 2010). Since sympathetic nerves are known to innervate ventricular myocardium, and elicit changes in the potassium and calcium channels, both of which are important components of the cardiac action potential, it is possible that oscillations in APD may also occur at this slower frequency (Zipes and Jalife, 1999; Workman, 2010; Taggart et al., 2011; Shen and Zipes, 2014). Such time-varying and regional oscillation of APD could have important implications for arrhythmogenesis, since beat to beat variability in APD has been associated with ventricular arrhythmogenesis and sudden cardiac death (Nearing and Verrier, 2002; Thomsen et al., 2007; Qu et al., 2010; Heijman et al., 2013; Xie et al., 2014). We have previously reported oscillations of ventricular action potential duration (APD) in humans occurring at free and voluntarily-controlled respiratory frequencies (Hanson et al., 2012). These data in subjects whose heart rate was paced at a fixed rate indicated that the variability in APD was independent of respiration-induced changes in heart rate. However, to the authors' knowledge, variation of APD in synchronization with Mayer waves has not been reported.

In order to study this relationship we recruited patients with heart failure in whom a biventricular pacing resynchronization device had been implanted. This enabled ambulatory recording of electrophysiology to be made directly from the left ventricular epicardium. As mental stress is known to enhance Mayer wave oscillations in blood pressure (Lucini et al., 2002), we devised a protocol whereby subjects were studied while watching emotionally-charged movie clips to elicit and maintain a state of arousal. A unipolar electrogram (UEG) was recorded from an epicardial electrode of the device and activation-recovery intervals (ARI) were obtained as a surrogate measure of APD. We observed oscillatory behavior of APD (ARI) in synchrony with respiration and blood pressure Mayer waves.

## Materials and methods

Ethical approval: The study was approved by the local Ethics Committee (Ref: 05/Q0702/89) and conformed to the standard set by the Declaration of Helsinki (latest revision: 59th WMA General Assembly). Written informed consent was obtained from all subjects.

### Subjects and protocol

Studies were performed in 14 ambulatory (outpatient) subjects with heart failure (all male, age 48–80 (Table 1). All subjects were undergoing treatment via implanted bi-ventricular cardiac resynchronization devices, which had been implanted for at least 6 months prior to study. Beta-adrenergic blocking agents were discontinued for 5 days prior to the study. Recordings were made with the subjects seated upright and stationary, facing a large display screen (approximately 1 m diagonal width, at a distance of 2.2 m) wearing over-the-ear headphones in a quiet room with dimmed lighting. To elicit and maintain heightened arousal of the sympathetic nervous system throughout the duration of the investigation, subjects were presented with excerpts containing dramatic sequences from the psychological horror film “The Shining” (Kubrick, 1980). Three excerpts were chosen, allowing three physiological recordings as described in Section Physiological Recordings. This study did not attempt to identify physiological changes in response to the specific stimuli, which are highly subjective.

**Table 1:** **Subject characteristics**.

**Subject**	**Age (years)**	**Gender**	**Diagnosis**	**NYHA Class**	**Ejection fraction (%)**
1	77	M	NICM	2	23
2	67	M	NICM	2	30
3	69	M	IHD	1	35
4	77	M	IHD	3	30
5	61	M	IHD	2	55
6	63	M	NICM	2	45–50
7	63	M	IHD	1	45
8	68	M	IHD	2	40
9	67	M	NICM	1	37
10	80	M	IHD	2	40
11	72	M	NICM	2	65
12	80	M	NICM	1	60–65
13	48	M	NICM	2	39
14	59	M	IHD	1	49
Range	48–50			1–3	23–65
Mean ± *SD*	68 ± 9				42 ± 12

*IHD, Ischemic heart disease; NICM, non-ischemic cardiomyopathy*.

### Physiological recordings

APD is strongly dependent on the interval between beats, which fluctuates over a wide range of frequencies from 0 up to half the heart beat frequency (i.e., every other beat); in this study, that confounding variation was isolated by maintaining the subjects' heart rate at a constant rate by right ventricular pacing (Hanson et al., 2012; Child et al., 2014), using their implanted pacing device. The pacing rate was chosen as a minimum rate sufficient to maintain continuous capture. Recordings were made after a minimum adaptation period of 2 min of pacing.

The implanted cardiac resynchronization device was also used to record unipolar electrograms from the left ventricular epicardial lead, sampled at 512 Hz. For a comparison between the unipolar electrogram and monophasic action potential. The devices used in this study were able to store five separate recordings of 30 s duration; a continuous recording of approximately 100 s was constructed by overlapping the 30 s recordings. Activation-recovery intervals (ARI) were measured from the time of minimum dV/dt of the electrogram QRS complex, representing local activation time, to the time of maximum dV/dt of the subsequent T-wave, representing local repolarization time (Wyatt et al., 1981; Haws and Lux, 1990; Coronel et al., 2006; Potse et al., 2009). The time resolution of ARI measurements was 1.95 ms (1/512 Hz). Ectopic beats occurred infrequently (median 0.68% of beats across all recordings) and were removed from analysis together with the successive beat. Afterwards, linear interpolation was applied to fill in missing ARI values (Task Force of the European Society of Cardiology and the North American Society of Pacing and Electrophysiology, 1996). Recordings in which ectopic beats comprised more than 10% of the total number of beats were rejected from the analysis (only 1 recording in this study).

Breathing activity was recorded by measuring chest circumference: a custom-adapted tension sensor (adapted from a RESPeRATE device, InterCure Inc., New York, NY, USA) fixed to an expandable elastic band was placed around the subject's abdomen. Tension in the elastic band was directly proportional to circumference, and hence inspiration. The signal was digitized and sampled at 6 Hz.

Arterial blood pressure was measured non-invasively using a finger cuff (Finometer pro, Finapres Medical Systems B.V., Amsterdam, The Netherlands). The signal was digitized and sampled at 1 kHz. Systolic blood pressure for each beat was computed as the maximum blood pressure measured from the pressure waveform using a script written in MATLAB (Mathworks, Inc., Natick, MA, USA).

These physiological recordings were synchronized using a short-duration electrical “spike” signal which was recorded across all measurement systems.

### Analysis of data

#### Respiratory frequency oscillation

**Figure 1:**
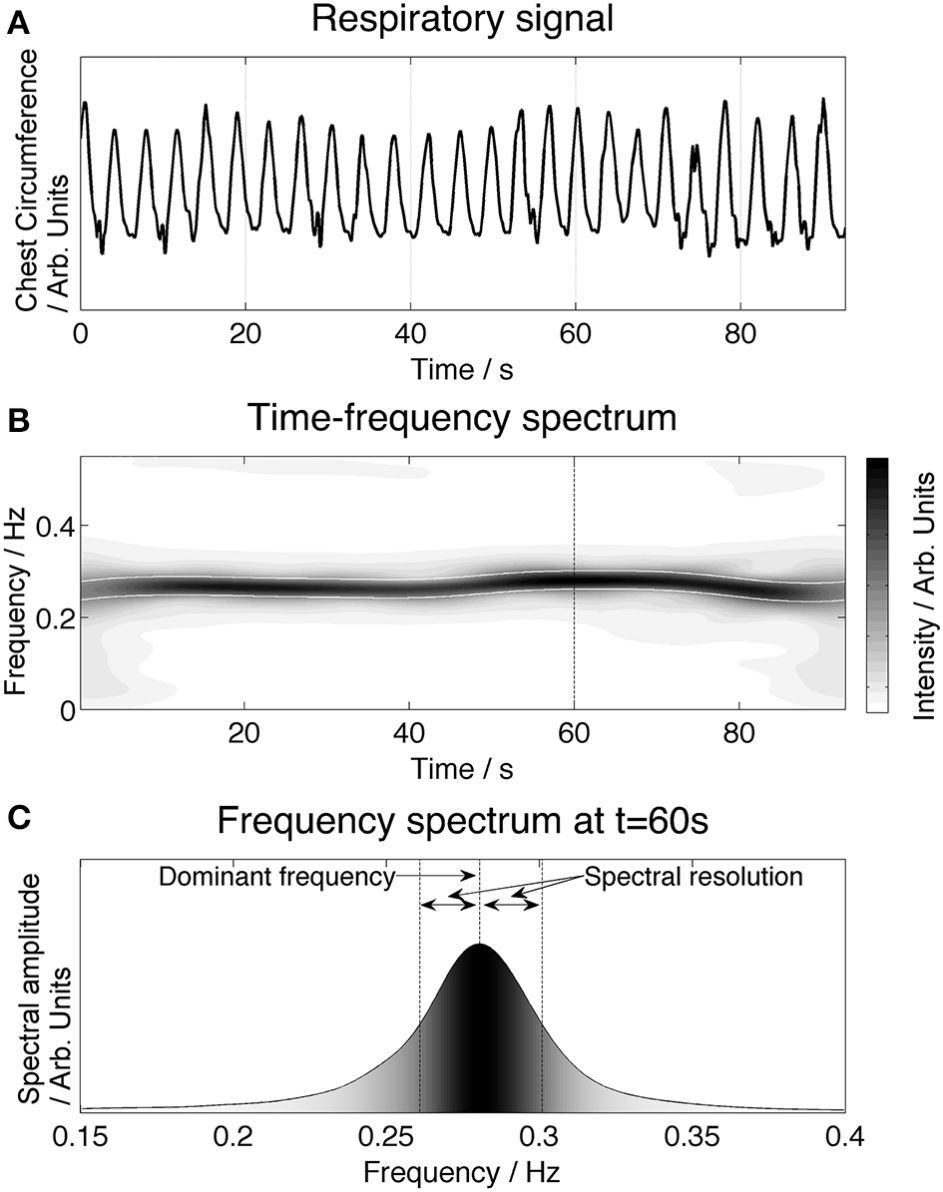
**Illustration of time-frequency analysis of the respiratory signal and the definition of the respiratory frequency band**. The upper panel **(A)** shows the time series of the respiratory signal. The corresponding time-frequency spectrum is presented in the middle panel **(B)**. The intensity of a specific frequency is given by the grayscale (white: low intensity, black: high intensity). An enhanced frequency band is clearly visible at the respiratory frequency (approximately 0.28 Hz in this example). The frequency spectrum **(C)** provides a cross-section of the time-frequency plot at 60 s, shown by a dashed line in **(B)**. The respiratory frequency band is defined by taking the maximum amplitude frequency ± the spectral resolution.

To quantify the respiratory frequency over the duration of the recordings we applied time-frequency analysis, which computes the frequency spectrum as a function of time. In Figure 1, the upper panel **(A)** shows the time series of the respiration signal. In the middle panel **(B)**, the time-frequency representation is shown: the horizontal axis represents time, the vertical axis gives the frequency and the amplitude is visualized by the grayscale. A high intensity (dark color) band is found around the respiratory frequency (approximately 0.28 Hz). The lower panel (C) demonstrates how the respiratory frequency band is defined as the dominant frequency ± the spectral resolution inside a high frequency band (0.15–0.5 Hz).

The time-frequency method used in this study is based on Cohen's class of quadratic time-frequency distributions, the smoothed pseudo-Wigner-Ville distribution (SPWVD). Previous research has demonstrated that this method provides good temporal and frequency resolution and is suitable to study cardiovascular interactions (Orini et al., 2012a). The spectral resolution depends on the SPWVD smoothing function, and was defined by the width of the smoothing function at the level of 50% of the maximum amplitude (Figure 1C).

To test whether ARI was oscillating with respiration, we evaluated if the ARI and respiration signals were coupled at the respiratory frequency. Coupling was studied using time-frequency coherence, Equation 1:

(1) $$\gamma (t,f)=\frac{\left|S_{xy}(t,f)\right|}{\sqrt{S_{xx}(t,f)S_{yy}(t,f)}}\gamma (t,f)\in [0,1]$$

*where*: γ(*t,f*) quantifies the strength of the linear coupling between signals *x* and *y* at time, *t*, and frequency, *f*. The strength of the coupling is defined between 0 (absence of correlation) and 1 (complete correlation). *S_xx_* and *S_yy_* are the time-frequency (TF) spectra of the *x* and *y* respectively (from the autocorrelation of each signal), and *S_xy_* is the cross-time-frequency spectrum, which is the TF spectrum of the cross-correlation between *x* and *y*. The cross-correlation evaluates the similarity between the two signals.

#### Mayer-wave frequency oscillation

In the second part of the analysis, the ARI signals were examined for oscillations at Mayer-wave frequencies. The relationship between Mayer waves in blood pressure and slow oscillations in ARI was studied as follows:

1. First, the blood pressure signals (BP) were analyzed for presence of Mayer waves. Mayer waves were assumed to be present in the signal if the average frequency spectrum contained a significant peak (see following Section Determination of Statistical Significance) in the low frequency band (0.04–0.15 Hz). The Mayer frequency was then defined as the peak-frequency ± the spectral resolution of the average frequency spectrum.
2. Time intervals were identified in which oscillatory behavior in ARI and/or blood pressure was statistically significant at the Mayer frequency.
3. The obtained intervals were categorized into the four possible conditions depending whether Mayer waves were present in BP and/or ARI measures:

**Table:** 

**Condition**	**BP**	**ARI**
1	×	×
2	×	✓
3	✓	×
4	✓	✓

*✓Presence of Mayer waves in the signal; × Absence of Mayer waves*.

A final step was applied in condition 4: to investigate whether the Mayer-wave oscillations in BP and ARI were significantly coupled. This was assessed by the TF coherence method described previously.

#### Determination of statistical significance

To determine the average peak to peak amplitude of the significant ARI oscillations we computed the amplitude spectrum and measured the peak at the respiratory and Mayer frequency.

The time frequency spectra contain components of signals across the frequency range, including some measurement noise, and it is important to identify whether the magnitudes of oscillations at respiratory and Mayer wave frequencies are significant in comparison to the noise. The method involves creating surrogate data (Faes et al., 2004): the samples of the measured signals were randomly shuffled to create a surrogate signal having the same important features at equal magnitudes while being completely uncoupled. This process was repeated 10,000 times to obtain a distribution of time frequency spectra and time frequency coherence values created by random. Consequently, the values of the real data signals were assumed to be significant if they exceeded a threshold set at the 100(1-α) percentile of the noise distribution, where α is the significance level of the statistical test. In this study, the threshold for significance was set at α = 5%.

## Results

Due to the challenging logistics of recording from multiple measurement systems concurrently it was not possible to achieve complete recordings in every instance, however recordings were obtained from 8/14, 13/14, and 14/14 subjects for the three recording periods, respectively. One electrophysiological recording was not analyzed because of the presence of multiple escape—un-paced—beats. One respiratory recording was rejected because it did not show a clear respiratory component. In one subject blood pressure measurements were not obtainable. A total of 33 ARI, 30 blood pressure, and 32 respiratory traces obtained from 14 subjects were analyzed. The average paced heart rate was 85 bpm.

### ARI oscillations at respiratory frequency

**Figure 2:**
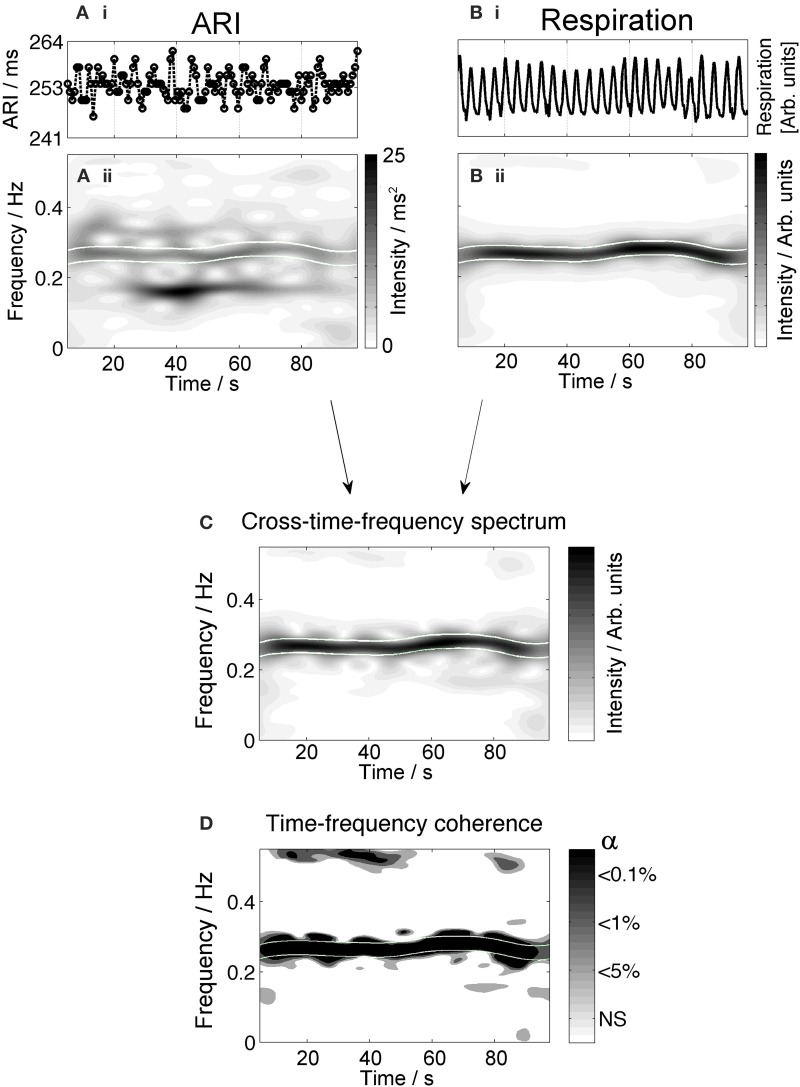
**Example plot showing oscillations in the ARI signal at the respiratory frequency**. The top graphs **(Ai,Bi)** show the time series of ARI and respiration. The corresponding time-frequency spectra are plotted below, **(Aii,Bii)**. The high intensity band in the time-frequency spectrum of the respiratory signal **(Bii)** represents the frequency of the respiratory signal (approximately 0.25 Hz). High intensity is also seen in the ARI time-frequency plot **(Aii)** in this frequency band. Oscillation is also present at other frequencies and times across the spectrum. The cross time- frequency spectrum **(C)** shows that the ARI and respiratory signal are correlated at the respiratory frequency and the other variations in each signal are not correlated. The results of coherence analysis in the lower panel **(D)** show the coherence at the respiratory frequency is significant at *p* < 0.001, indicating that both signals are coupled at this frequency. *NS* = not significant.

All patients showed significant ARI oscillation at the respiratory frequency (Table 2). The average ARI peak to peak differences per patient were between 2.2 and 6.9 ms and averaged 5 ms. Figure 2 shows an example in which ARI oscillations at the respiratory frequency were observed during the entire recording period. The two uppermost plots, **Ai** and **Bi** show the time series of ARI and respiration respectively. The ARI exhibits cyclical variation at a frequency similar to the respiratory pattern, notably in the absence of respiratory sinus arrhythmia since the heart rate is paced (at 80 bpm in this case). Plots **Aii** and **Bii** show the corresponding time-frequency spectra. The ARI time-frequency spectrum shows an enhanced amplitude (higher intensity) at the respiratory frequency band (approximately 0.25 Hz) indicating that the ARI is oscillating at this frequency for the duration of the recording (in addition to transitory oscillations at other frequencies). The cross-time-frequency spectrum (plot C) shows a high intensity inside the respiratory frequency band which demonstrates a correlation between ARI and respiration at the respiratory frequency. The time-frequency coherence analysis (plot D) shows that the coupling between ARI and respiration is statistically significant at the respiratory frequency for the whole length of the recording. In all other subjects coupling between ARI and respiration occurred intermittently during the recordings. The average coupling time was about 43% of the total recording time for each subject.

**Table 2:** **Oscillatory behavior of ARI**.

	**Oscillations at respiratory frequency**			**Oscillations at Mayer frequency**		
**Subject**	**Coupling**	**Amplitude, ms**	**Duration, % of total recording**	**Coupling**	**Amplitude, ms**	**Duration, % of total recording**
1	+	5.7	9	−	−	−
2	+	2.3	57	−	−	−
3	+	3.5	27	−	−	−
4	+	6.9	40	−	−	−
5	+	3.9	61	−	−	−
6	+	3.6	42	+	5.5	19
7	+	4.4	95	−	−	−
8	+	3.5	36	−	−	−
9	+	2.7	52	+	2.9	8
10	+	2.2	34	+	5.4	33
11	+	5.2	21	+	3.6	66
12	+	4.4	16	−	−	−
13	+	2.8	68	X	9.2*	X
14	+	2.4	42	+	3.4	18
Range		2.2–6.9	9–95		2.9–9.2	8–66
Mean ± SD		3.8 ± 1.4	43 ± 23		5.0 ± 2.3	29 ± 23

In all subjects, ARI showed oscillations at the respiratory frequency. In 6 subjects we observed significant slow oscillatory behavior in the Mayer frequency range, which was coupled in 5 subjects with Mayer waves in blood pressure (BP not available in 6th subject). The amplitude quoted is the average peak to peak amplitude over the total duration of oscillation for each subject. Duration represents the total period for which oscillation was both of significant magnitude (p < 0.05) and coupled to oscillation in BP, expressed as a fraction of the total duration of the three recording periods. SD, standard deviation; x, data not available. ^*^, Blood pressure recordings not available; –, no significant oscillation recorded; +, coupling significant at *p* < 0.05.

### ARI oscillations at mayer-wave frequencies

**Figure 3:**
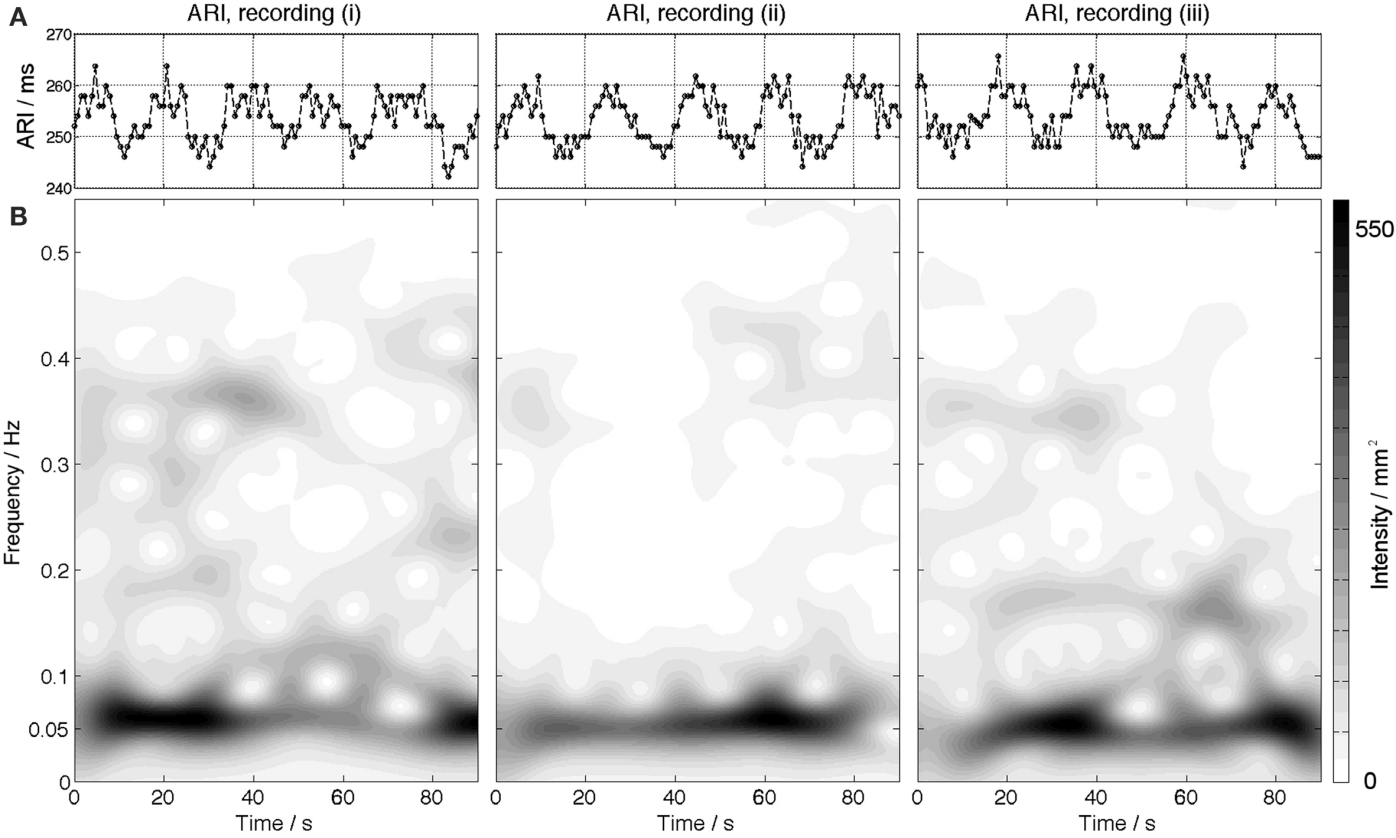
**An example is shown for one subject illustrating ARI time series (A) oscillating with a peak-to-peak amplitude of 10–15 ms**. The lower panels **(B)** show the corresponding time-frequency spectra. The spectra show an increased intensity at a frequency of 0.05 Hz.

Significant Mayer waves were observed in all patients in whom blood pressure recordings were available, (13/14). Oscillatory behavior—of ARI at the Mayer wave frequency was observed in 6/13 (46%) of subjects (Table 2). The average peak to peak ARI differences ranged from 2.9 to 9.2 ms between subjects. An example is shown in Figure 3 in which ARI oscillates at a frequency of 0.05 Hz and has a maximum peak-to-peak amplitude of approximately 15 ms.

**Figure 4:**
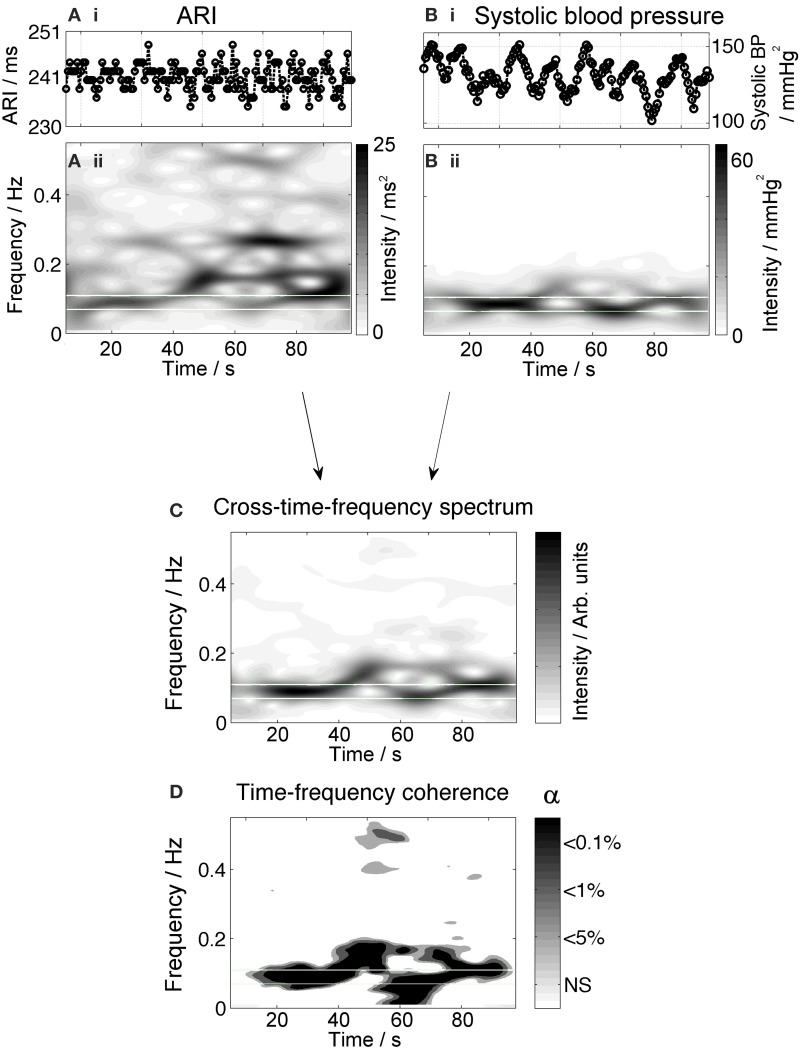
**Example measurements from one subject showing oscillatory behavior of ARI at the Mayer frequency**. The upper panels **(Ai,Bi)** show the time series of ARI and blood pressure, respectively. Blood pressure shows prominent oscillation with a 10-s period; ARI shows oscillation at this rate as well as variation at higher frequencies. The corresponding time-frequency spectra, **(Aii,Bii)**, show high-intensity bands highlighting the presence of waves at a Mayer wave frequency (0.1 Hz). The cross time-frequency spectrum **(C)** demonstrates that the ARI and blood pressure are correlated at the Mayer frequency and not at other frequencies of oscillation. The results of coherence analysis in the lower panel **(D)** show the coherence at the respiratory frequency is significant at *p* < 0.001, indicating that both signals are coupled at this frequency over most of the recording.

Synchronization of low-frequency oscillations in ARI and Mayer waves is shown in Figure 4. Figures 4Ai,Bi show the time series of ARI and systolic blood pressure with clearly-visible waves in the blood pressure occurring at a 10 s period. The time frequency spectrum of this signal, Figure 4Bii, shows a high intensity at 0.1 Hz. The ARI time-frequency spectrum Figure 4Aii also shows increased intensity at 0.1 Hz. The cross-time-frequency spectrum, Figure 4C, confirms a correlation between ARI and systolic blood pressure at this frequency. Finally, coherence analysis shown in the lower panel, Figure 4D, demonstrates that the slow oscillations in ARI are significantly coupled with oscillations in systolic pressure, throughout 78% of the recording time (average: 66% over all 3 recordings for this subject, summarized in Table 2).

When Mayer waves were present in BP, significant ARI oscillations in the Mayer frequency range were observed an average of 29% of the time, across all recordings and patients. On occasions when oscillations of ARI and systolic pressure were both present, they were coupled at the significance level of *p* < 0.05 for 75–100% of the duration of the oscillatory period. When Mayer waves were absent in blood pressure, there were some instances of significant ARI oscillations in the Mayer frequency range; across all recordings and patients this occurred for 8% of the time. Data for all patients are summarized in Table 2.

There was no relation between ejection fraction (EF) or heart failure class and the occurrence of slow oscillating behavior in ARI that was correlated with Mayer waves. However, five of the six patients that showed slow oscillating behavior had non-ischemic cardiomyopathy and only 1 had ischemic heart disease (IHD). None of the other patients with IHD showed these slow oscillations.

## Discussion

Ambulatory heart failure patients exhibited oscillation of left ventricular epicardial APD (measured as ARI) at two main frequencies. Oscillations in APD were present for all subjects at the respiratory frequency which were strongly correlated with respiration. Oscillations in APD were also present at a slower frequency (approximately 0.1 Hz) in a proportion of subjects which were coupled with systolic blood pressure oscillations. These APD oscillations were independent of beat to beat interval, which was constant (paced).

Ventricular activation exhibits cyclical variation such that the interval between heartbeats varies with the respiratory cycle, increasing with expiration and decreasing with inspiration, known as respiratory sinus arrhythmia (Anrep et al., 1936; Cohen and Taylor, 2002; Eckberg, 2009). It was recently reported that ventricular APD (measured as ARI) also varies cyclically with respiration (Hanson et al., 2012). This study in subjects with normal ventricles examined 10 left and 10 right ventricular endocardial sites at breathing frequencies of 6, 9, 12, 15, and, 30 breaths per min. Cyclical variation of APD at the respiratory frequency was observed with maximum magnitudes over a range from 0 to 26 ms. The present observations corroborate the existence of APD oscillations in humans and extend the findings to ambulatory patients with heart failure.

Oscillations in arterial pressure have long been known to occur at a frequency slower than respiration, known as Mayer waves (Mayer, 1876; Julien, 2006; Malpas, 2010). The present results demonstrate for the first time measurements of ventricular APD oscillations at the frequency range of the known Mayer wave oscillations, which were observed in a paced, heart-failure human model. Although commonly occurring at a frequency of approximately 0.1 Hz, Mayer waves occur over a fairly wide range of frequencies spanning the range of 0.03 to 0.15 Hz (Cohen and Taylor, 2002). The frequency range of the slow oscillations we observed was 0.04 to 0.12 Hz.

### Experimental model

The methodology employed in this study was novel: the study was designed to enable measurements of epicardial ventricular APD in ambulatory humans during a period of enhanced emotional arousal. The left ventricular pacing electrode of the biventricular pacing device enables recordings to be made of UEGs from the epicardium, while steady-state pacing was maintained from the right ventricular electrode in order to isolate changes in ARI (APD) from cycle length-dependent effects.

Mayer oscillations are associated with oscillations of sympathetic nervous tone (Cevese et al., 2001; van de Borne et al., 2001): these subjects were studied while seated in an upright position which is known to facilitate sympathetic activation. Sympathetic activity may have been further exaggerated in this heart failure patient group, whose hemodynamic function is less than normal, particularly during the pacing strategy employed in this experiment. Emotional arousal was enhanced by the use of movie excerpts which are considered to be among the most-powerful stimuli to elicit affective responses in the laboratory setting (Westermann et al., 1996; Schaefer et al., 2010). In this study we made no attempt to investigate how oscillatory APD behavior changes in response to different degrees of stress and tranquility, which is a highly-subjective measurement, but focussed on establishing the existence of these phenomena under conditions likely to enhance their presence.

The time frequency coherence method applied in this study accounts for the time-varying nature (non-stationarity) of the oscillations in ARI, systolic blood pressure and respiration signals. It has been demonstrated recently that the Cohen's class distributions used for this study can be used to reliably quantify the dynamic interactions between cardiovascular signals, such as heart rate variability, BP and respiration (Orini et al., 2012a,b). The statistical significance of the time frequency coherence was assessed by using surrogate data analysis to establish the “noise floor” of the recordings to determine the confidence interval. Subsequently it was demonstrated that oscillations in ARI and BP showed significant coupling at the respiratory rate and at Mayer wave frequencies with significance levels reaching *p* < 0.001 for periods.

In this study, we examined whether slow oscillatory behavior in both APD and blood pressure was present or absent at the same time. The results suggest that if Mayer waves are absent in blood pressure, oscillations in APD at the Mayer wave frequency are also likely to be absent. On the other hand, when Mayer waves were observed in blood pressure, they were sometimes but not always accompanied by coupled oscillations in APD. It should be noted that since APD variation may be heterogeneous, it is possible that APD oscillation was present in ventricular regions which were not captured by the single-site epicardial recording used here (see Limitations).

### Underlying mechanisms

#### At the respiratory frequency

These results support a relationship between respiration and electrophysiology, demonstrating significant coupling between oscillation in ARI and BP with respiration. A previous study employed a constant-rate breathing protocol to observe rate-dependence and phase relationships (Hanson et al., 2012); in this study there was natural variation in each subject's breathing rate and statistical coupling was retained. We have previously proposed and discussed in some detail several possible mechanisms as underlying the APD oscillations seen at the respiratory frequency in subjects with normal ventricles (Hanson et al., 2012). In brief: One possibility is mechano-electric feedback whereby changes in ventricular load alter the electrophysiology (Kohl et al., 2006; Taggart and Sutton, 2011). Respiration results in a cyclical change in ventricular filling and hence in myocardial loading conditions (Guz et al., 1987). Furthermore, these effects are more pronounced when cycle length is maintained constant by constant pacing as was the case in the present study (Innes et al., 1987). Previous studies in humans have shown that altering ventricular loading alters ventricular APD over a range comparable to that seen in the present study (Taggart and Sutton, 2011). Two other mechanisms which have been proposed to account for respiratory related oscillations of sinus node firing could be operative (Task Force of the European Society of Cardiology and the North American Society of Pacing and Electrophysiology, 1996; Malliani, 2000; Cohen and Taylor, 2002; Eckberg, 2009; Karemaker, 2009). The baroreflex mechanism attributes fluctuations in RR interval to baroreflex-induced fluctuations in vagal activity in response to respiration induced changes in stroke volume (de Boer et al., 1987; Innes et al., 1993). An alternative mechanism is central gating of autonomic drive to the myocardium by central respiratory networks (Spyer and Gilbey, 1988; Dergacheva et al., 2010). In this heart failure model we observed similar magnitudes of variation in ARI as those observed in normal ventricles (Hanson et al., 2012), however this was only a single-point measurement and it is likely that ARI variation was heterogeneous across the myocardium as previously observed. Multi-site mapping is recommended to identify whether diseased ventricles exhibit a higher-degree of local heterogeneity in ARI as a result of respiratory-related variation.

#### At mayer-wave frequencies

The presence of significant coupling between ARI and systolic blood pressure suggests the possibility that both signals may be driven by a common source. That possibility is further supported by occurrences of significant oscillations at Mayer frequencies in ARI in the absence of oscillations in BP, and vice-versa. The data do not wholly support a model of ARI oscillation being dependent on BP oscillation, nor vice-versa. The experimental model was not able to identify phase relationships between oscillations in ARI and BP since the periods of oscillation and coupling varied and steady-state oscillation was not observed. Mayer wave oscillations on systolic blood pressure are generated by oscillations of sympathetic tone (Cevese et al., 2001; van de Borne et al., 2001). Two mechanisms have been proposed to explain their consistent frequency. One theory attributes the rhythmicity of Mayer waves to pacemaker-like activity of an oscillator in the brainstem or spinal cord region generating sympathetic nerve activity. An alternative theory proposes that Mayer waves are oscillatory responses to hemodynamic changes and governed by the magnitude of the hemodynamic change and the sensitivity of the sympathetic limb of the baroreflex (for review see Julien, 2006 and Malpas, 2010). The mechanism underlying the synchronous slow oscillations in APD has yet to be determined. Either of the above mechanisms could be associated with phasic autonomic input to the ventricular myocardium and a direct effect on the cellular electrophysiology. Our study does not allow discrimination between the two possibilities. Mechano-electric feedback may play a role whereby the APD oscillations are secondary to the phasic mechanical stimuli accompanying the oscillations in systolic pressure. In patients with heart failure the myocardial stress may be different to that in normal hearts because of altered diastolic stiffness. Further work is recommended to elucidate the mechanisms.

### Limitations

In this study, it was not possible to pace and record from the same lead due to electrical interference, hence the RV was paced and LV sensed. This pacing protocol may have significantly affected the haemodynamic efficiency. In our previous study frequency in subjects with normal ventricles, we observed that the presence and magnitude of APD oscillation on the endocardium at the respiratory was inhomogeneous (Hanson et al., 2012). However, the electrophysiological measurements in the present study were obtained from single site recordings on the left ventricular epicardium, and therefore we cannot comment on whether the oscillatory behavior we observed on APD was a local or generalized phenomenon. In our previous study in which blood pressure was recorded directly from the aorta, we performed phase analysis between BP and APD; this was not possible in the present study owing in part to the technical limitations of the non-invasive system (Finometer pro, Finapres Medical Systems B.V., Amsterdam, The Netherlands). Intra-arterial measurements were precluded in this set-up as they were not appropriate for this ambulatory patient group. The question as to whether slow wave APD oscillations are confined to heart failure patients or also occur in normal subjects cannot be addressed at the present time owing to the inability to perform comparable intracardiac electrophysiology measures in normal subjects.

### Implications

In general, oscillatory behavior of APD such as APD alternans or beat to beat APD variability, reflect repolarization instability and an increased susceptibility to arrhythmogenesis (Nearing and Verrier, 2002; Thomsen et al., 2007; Qu et al., 2010; Heijman et al., 2013; Xie et al., 2014). It remains to be determined whether the APD oscillation we observed, particularly at the slower frequencies, represents simply a benign enhancement of normal physiology or whether it represents a destabilization of the repolarization process which may have consequences in the context of arrhythmogenesis.

## Author contributions

Ben Hanson, Peter Taggart, Nick Child and Jaswinder Gill conceived and designed the experiments. All authors took responsibility in collecting, analyzing and interpreting the data, with particular individual input in the following areas: electrophysiology (Peter Taggart), analysis (Stefan Van Duijvenboden), experimentation (Nick Child). All authors contributed to drafting or revising the manuscript and all authors approved the final version of the manuscript.

### Conflict of interest statement

The authors declare that the research was conducted in the absence of any commercial or financial relationships that could be construed as a potential conflict of interest.
